# Case of Pulp Regeneration Following Autologous Transplantation of an Incompletely Rooted Tooth

**DOI:** 10.1155/crid/7121013

**Published:** 2025-04-08

**Authors:** Hiroyuki Kimura, Tsuyoshi Kumano, Taro Eida

**Affiliations:** Kimura Dental Clinic, Kumamoto City, Japan

**Keywords:** autologous tooth transplant, case report, pulp regeneration, root incomplete tooth

## Abstract

Autologous tooth transplantation is a procedure that involves the replacement of a natural tooth with another at a new site or a surgically created recipient wound. With an appropriate selection of indications, studies report over 90% survival rates for transplanted teeth, even after 10 years. In this report, we presented a case of an incomplete rooted maxillary right third molar that was transplanted into the mandibular right first molar area. We also evaluated the stability of periodontal tissue, root development, and pulp regeneration after autologous tooth transplantation. The patient was a 20-year-old female who presented with the chief complaint of caries in the right mandibular first molar. The affected tooth was diagnosed as suitable for extraction because the crown decay progressed from the subgingival region to the furcation. Autologous tooth transplantation was performed using a right maxillary third molar with an incomplete root as the donor's tooth. After 6, 9, and 11 years of follow-up, radiographs showed stable periodontal tissue and a root apical condition. Root formation in the transplanted tooth proceeded smoothly without any problems. Root canal treatment was not required because the pulp demonstrated signs of vital response, and no stenosis or calcification of the pulp cavity was observed. The transplanted tooth also had a satisfactory occlusal function. Autologous tooth transplantation is a useful option for occlusal reconstruction following tooth extraction.

## 1. Introduction

Fixed prosthetic procedures or dental implants are options for the occlusal restoration of a single molar. However, as mentioned in references [1, 2], autologous tooth transplantation may be a potentially valuable but overlooked treatment option for occlusal reconstruction if a suitable donor tooth is available [3, 4].

Autologous tooth transplantation has several advantages over fixed bridges or dental implants. Notable among them is its applicability to adolescents and young adults with incomplete maxillofacial growth [5]. Moreover, the transplanted tooth has the potential to stimulate alveolar bone growth as a result of the presence of the periodontal ligament. Additionally, if necessary, the transplanted tooth can be moved to its ideal position through orthodontic treatment [5, 6]. Another advantage is the possibility of pulp tissue regeneration, which eliminates the need for root canal treatment. However, the factors that influence the success of autologous tooth transplantation must be carefully considered, for example, the surgical skill and knowledge of the operator, patient selection, local inflammatory condition of the recipient site, need for endodontic treatment, and availability of periodontal ligament tissue on the donor tooth and recipient site [1, 7–9]. Furthermore, a systematic review by Machado et al. included studies with a follow-up period of 6 years or more to analyze long-term prognosis [7]. The meta-analysis revealed a survival rate of 81%, indicating an excellent long-term treatment prognosis for autologous tooth transplantation, as discussed in previous studies [10, 11]. Moreover, if the pulp regenerates, root canal treatment is unnecessary, which is advantageous for autologous tooth transplantation.

In this report, we present a case of an incomplete rooted maxillary right third molar that was transplanted into the mandibular right first molar location. In addition, we observed the stability of the periodontal tissue, root development, and pulp regeneration after autologous tooth transplantation.

## 2. Case Presentation

### 2.1. Patient

**Figure 1:**
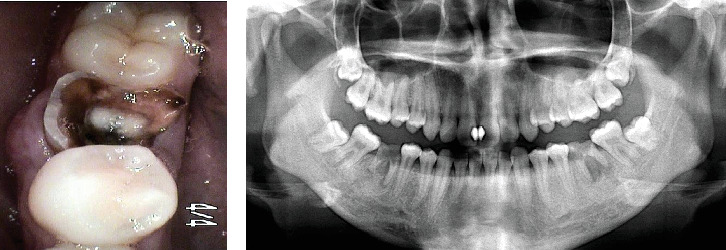
(a) A clinical view of the mandibular right first molar at the first examination. (b) A panoramic radiograph at the first examination.

A 20-year-old female with no systemic medical history visited our dental clinic complaining of caries of the mandibular right first molar. Upon examination, the affected tooth had crown decay, which progressed from the subgingival area to the furcation, causing swelling of the buccal gingiva (Figure 1a).

Panoramic radiography revealed a crown collapse of the affected tooth and a radiolucent lesion extending from the root furcation to the root apex. In addition, the maxillary right third molar, which had a relatively conical and incomplete root, was impacted (Figure 1b).

The patient refused a fixed bridge or dental implant. Therefore, we proposed autologous tooth transplantation as an alternative treatment, and consent was obtained after a thorough explanation. The right mandibular third molar was excluded as a candidate donor tooth because of its curved root. Furthermore, a cone-beam CT (CBCT) scan revealed that the cross-sectional area of the extraction socket of the mandibular right first molar was larger than that of the right maxillary third molar of the donor tooth. Therefore, it was thought that it would be difficult to suture the gingival flap tightly after transplanting the donor tooth. Hence, we decided to wait for epithelialization of the extraction socket after tooth extraction.

### 2.2. Operation

**Figure 2:**
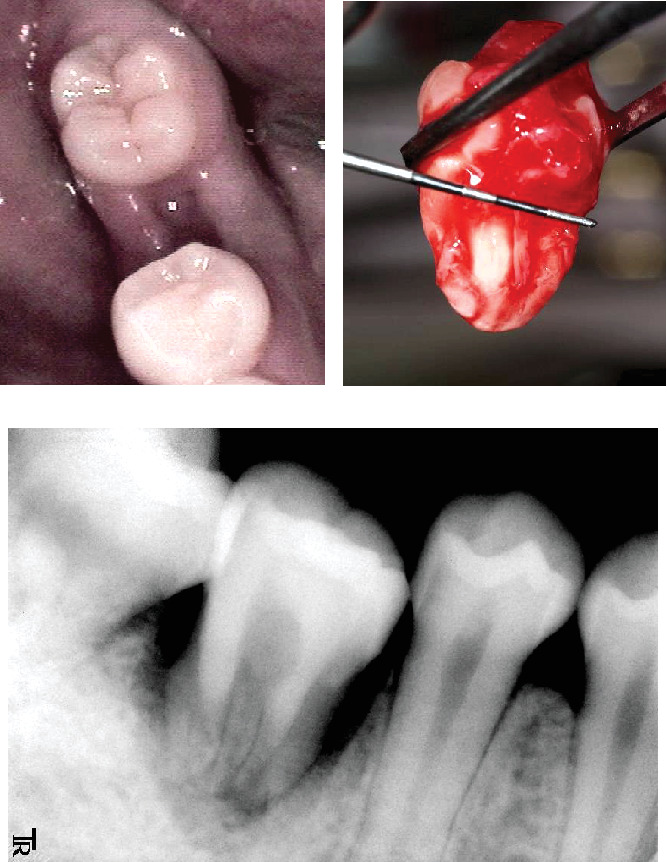
(a) A clinical picture of the recipient site after extraction at the transplantation. (b) The extracted maxillary right third molar for transplantation. (c) A radiograph taken at 2 weeks after the transplantation.

Fifty-two days after the extraction of the mandibular right first molar, donor tooth transplantation was performed under local anesthesia (Figure 2a,b). When extracting the donor tooth, we performed a gentle dislocation operation to avoid damaging the periodontal ligament as much as possible.

At the recipient site, an incision was made mesiodistally to the periosteum at the alveolar crest, and the gingival flap was turned over to expose the bone surface. The socket formation was performed using a handpiece equipped with a slow-speed (approximately 20,000 rpm) round bur under copious irrigation.

To ensure the proper placement of the donor's tooth when forming the socket, we referred to the distance from the cementoenamel junction of the donor's tooth to the root apex and the diameter of the section perpendicular to the tooth axis at the cementoenamel junction, which had been measured in advance using CBCT examination.

The extracted donor tooth did not move after insertion into the prepared socket. It was transplanted within 20 min using a cruciate suture with 4–0 silk strings. Tsukiboshi states that suture fixation is simple and advantageous, particularly because of the significant bleeding that typically occurs on the day of transplantation [12]. Additionally, Kogai et al. report that suture fixation provides better results in stabilizing the periodontal ligament [13].

The time required from the extraction of the donor tooth to transplantation was less than 20 min. A minimal occlusal adjustment was made to avoid occlusal contact between the donor and opposing teeth. The patient was instructed to take 200 mg of cefcapene pivoxil and 60 mg of Loxonin twice daily for 3 days. The silk sutures were removed after 1 week (Figure 2c).

### 2.3. Postoperative Course

Because of the incomplete root formation of the transplanted tooth, as noted by Tsukiboshi, it is essential to continuously monitor the tooth's progress to ensure normal healing and detect any complications that could lead to the loss of the autotransplanted tooth. In cases of incomplete root formation, pulp healing is expected; therefore, close monitoring during the first 3 months is crucial to prevent inflammatory resorption, and continued observation between 3 and 12 months is necessary to ensure pulp healing (obliteration of the pulp cavity) [14].

**Figure 3:**
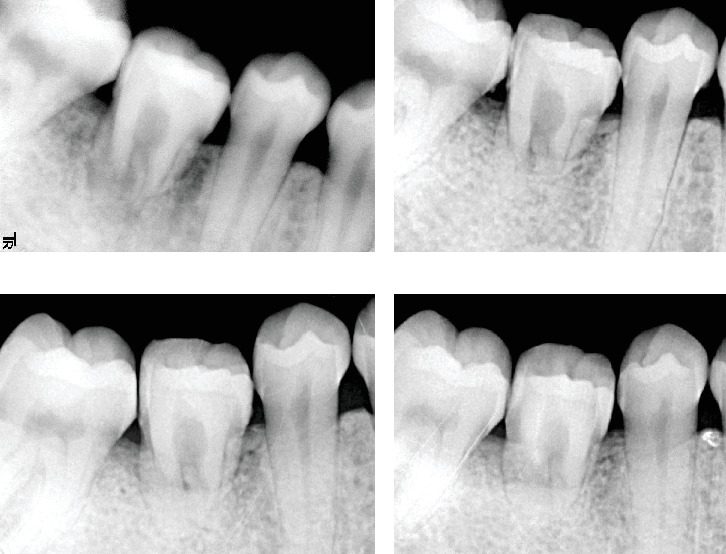
(a) A radiograph taken at 1 year after the transplantation. (b) A radiograph taken at 6 years after the transplantation. (c) A radiograph taken at 9 years after the transplantation. (d) A radiograph taken at 11 years after the transplantation.

The patient adhered to a maintenance program, attending follow-up visits every 3–6 months, with occlusal checks and adjustments as needed. The patient was also advised to undergo regular examinations and dental radiographs to assess root apex formation, alveolar bone regeneration, and changes in the pulp cavity. Radiographic examination 1-year posttransplantation showed regeneration of the alveolar bone with a maintained level. No obvious tooth root resorption was observed (Figure 3a). Thereafter, follow-up was interrupted for approximately 3 years for patient-specific reasons.

Radiographic examinations 6, 9, and 11years posttransplantation revealed no obvious resorption of the alveolar bone or changes in the alveolar level (Figures 3b, 3c, and 3d). Additionally, the periodontal ligament space was examined, and no tooth resorption or apical lesion formation was observed. Stenosis or calcification of the pulp cavity was not observed. An electric pulp test (EPT) was performed to determine whether the transplanted tooth retained its vital pulp. The transplanted tooth demonstrated a positive reaction during the test. The EPT is an electrical vitality test used to diagnose the health of the dental pulp and confirm whether it is alive [15].

The Periotest values measured 9 years posttransplantation presented a value of 24, whereas the most recent test indicated a Periotest value of 5. This value is close to that of natural teeth, suggesting that the periodontal ligament is healthy and the possibility of ankylosis is low. Furthermore, the depth of the gingival sulcus around the transplanted tooth was within 3 mm, and no bleeding was observed during probing.

**Figure 4:**
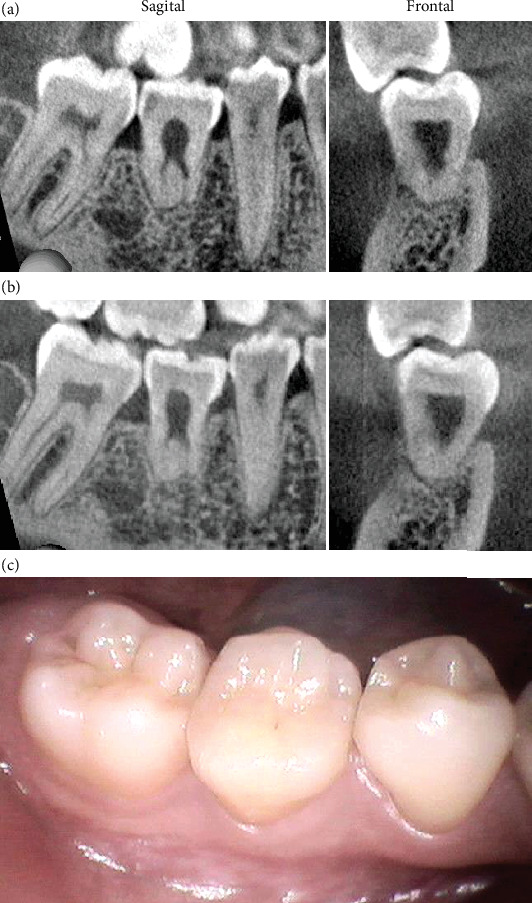
(a) CBCT appearance taken at 9 years after the transplantation. (b) CBCT appearance taken at 11 years after the transplantation. (c) A clinical picture of the transplanted tooth at 11 years after the transplantation.

CBCT scans taken at 9 and 11 years after transplantation illustrated complete root formation and alveolar bone regeneration, with no obvious tooth resorption or significant pulp cavity calcification (Figures 4a, 4b, and 4c).

The patient is currently following a maintenance program every 4–6 months and is clinically able to masticate without any problems. During the patient's visit to our clinic, we checked for occlusion and performed occlusal adjustments.

## 3. Discussion

This case report describes the progress of a 20-year-old female's transplanted tooth with an incomplete root over 11 years and the stability of the periodontal and pulp tissues.

To achieve sufficient closure of the transplanted tooth with a gingival flap, autologous tooth transplantation was performed in two steps after the epithelialization of the extraction socket. The patient progressed well without a root canal or prosthetic treatment. Avoiding prosthetic procedures may be advantageous in autologous tooth transplantation [16].

The donor tooth had incomplete root formation with conical root morphology. The success rate of autologous tooth transplantation was reported to be 94% in a group of donor teeth with an open apex and 84% in a group of donor teeth with apical closure [17]. Another study reported a success rate of 92% for donor teeth with half of the root formation [18]. Autologous transplantation, which is highly predictable, should be considered a treatment option when a suitable donor tooth is available.

If a tooth with an incomplete root is used as a donor, regeneration of the pulp of the donor tooth and root growth can be expected after transplantation [19–22]. Tsukiboshi described pulp healing as the regeneration of capillaries in the pulp cavity and subsequent pulp canal obliteration. In a tooth with an incomplete root, the Hertwig epithelial sheath remains, and capillary regeneration is thought to proceed through the primary apex surrounding this sheath. As the capillaries regenerate, pulp cells inside the epithelial sheath proliferate into the pulp cavity [20, 21], which is filled with living cells within a few months. However, calcification occurs rapidly, leading to pulp canal obliteration [23]. Moorrees et al. [24] investigated the percentage of pulp healing after transplanting 370 donor premolars at various stages of root development. Accordingly, pulp healing can be expected when up to four-quarters of the tooth root has formed and the apical foramen is wide open. Conversely, Andreasen et al. investigated the healing status of the dental pulp based on the diameter of the apex and found that if the diameter of the apical orifice is ≥ 1 mm, there is a 94% probability that the pulp can be expected to heal [23]. According to Lucas-Taule et al., donor teeth with open apices show fewer complications than those with closed ones and are considered the gold standard for transplantation [25].

In this case, even 11 years after transplantation, the donor tooth presented a positive reaction in the EPT, and no significant calcification was observed in the pulp cavity on CBCT.

Andreasen et al. reported root development in donor teeth after transplantation. They reported that regardless of the stage of root development of the donor tooth, most end up with partial development [20].

In this case, it was necessary to continue regular radiographic examinations and observe changes in the pulp cavity and root apex.

Autologous tooth transplantation procedures are simple. Lucas-Taule et al. reported that complications were lower in donor teeth with open apices compared with those with closed apices [25].

Unsuccessful autologous tooth transplantation is associated with excessive surgical invasiveness during the operation, patient age > 40 years, contaminated donor teeth, and selection of donor teeth with a periodontal pocket of > 4 mm [26]. The periodontal ligament is highly sensitive to changes in pH and osmotic pressure. Although most of the periodontal ligament can survive in a dry state for up to 18 min, more than half will die after 30 min, and approximately all will die after 120 min [27]. In this case, the donor tooth was outside the mouth in saline for less than 20 min.

The main reasons for the failure of autologous tooth transplantation are periodontal tissue attachment loss (54.9%), root resorption (26.5%), dental caries (4.0%), and tooth fracture (2.9%) [28]. In the present case, the patient was 20 years old at the time of the procedure and had no history of periodontal disease. A patient's general condition, age, oral hygiene, and high compliance, including regular clinical visits, play important roles in achieving good results.

The transplanted tooth also functioned well upon occlusion, indicating that autologous tooth transplantation could be a useful option for reconstructing occlusal function after tooth extraction.

Previously, I submitted another case report on autogenous tooth transplantation to Wiley: https://onlinelibrary.wiley.com/doi/10.1155/2021/5512804 [29]. The main similarities and differences between the previous case and the current case are as follows: The primary difference is the introduction of a CT scanner (Morita 3DX Multi Image Micro CT) approximately 20 years ago, which enabled precise assessment of the three-dimensional shape and size of the donor tooth. This advancement significantly reduced the time required for socket preparation. In contrast, in the previous case, the size and shape of the donor tooth could only be estimated using plain dental x-rays or panoramic tomography (Pan-Tomo) images. These findings highlight the importance of CT as a valuable diagnostic tool.

The fundamental procedures for autogenous tooth transplantation remained consistent between the two cases. A common approach in both cases was to minimize damage and degeneration of the periodontal ligament of the donor tooth. To achieve this, careful extraction techniques were employed, and efforts were made to minimize the time the donor tooth remained outside the oral cavity before socket preparation was completed. In both cases, the transplanted tooth was stabilized using sutures for approximately 1 week. However, a key difference was the necessity for root canal treatment in the previous case as a result of the complete development of the tooth roots. In contrast, in the current case, the donor tooth had incompletely developed roots, allowing for the monitoring of root formation using plain dental x-rays and CT scans. In this regard, CT imaging proved to be a highly useful tool for preoperative planning and postoperative evaluation.

## Data Availability

Data sharing is not applicable to this article as no new data were created or analyzed in this study.
